# Sustained onabotulinumtoxinA therapeutic benefits in patients with chronic migraine over 3 years of treatment

**DOI:** 10.1186/s10194-018-0918-3

**Published:** 2018-09-17

**Authors:** Michail Vikelis, Andreas A. Argyriou, Emmanouil V. Dermitzakis, Konstantinos C. Spingos, Nikolaos Makris, Evangelia Kararizou

**Affiliations:** 1 Headache Clinic, Mediterraneo Hospital, Glyfada, Greece; 2 Glyfada Headache Clinic, 8 Lazaraki Str, 16675 Glyfada, Greece; 3 Headache Outpatient Clinic, 1st Department of Neurology, National and Kapodistrian University of Athens, Athens, Greece; 4 Neurology Department, Saint Andrew’s State General Hospital of Patras, Patras, Greece; 5 Department of Neurology, “Geniki Kliniki” Euromedica, Thessaloniki, Greece; 6 Corfu Headache Clinic, Corfu, Greece

**Keywords:** Migraine, Chronic migraine, Long term treatment, Sustained efficacy, onabotulinumtoxinA, Botox®

## Abstract

**Background:**

Evidence on whether the therapeutic effect and good safety profile of onabotulinumtoxinA (Botox®) in chronic migraine (CM) patients is maintained over long term treatment is still limited. We herein aimed at assessing whether there is a sustained benefit and good safety with repeated onabotulinumtoxinA sessions in CM over more than three years of treatment.

**Methods:**

We prospectively enrolled 65 CM patients, who were classified as responders after three sessions of onabotulinumtoxinA and were eligible to further continue treatment. Data documenting longitudinal changes from the trimester after the third onabotulinumtoxinA administration (T1) to the trimester after completing two years of treatment (T2) and eventually to the trimester after completing three years of treatment (T3) in (i) mean number of monthly headache days (ii) migraine severity as expressed by the mean number of days with peak headache intensity of > 4/10, and (iii) mean number of days with use of any acute headache medication, were prospectively collected from patients’ headache diaries.

**Results:**

A total of 56 (86.1%) of 65 patients achieved to attain onabotulinumtoxinA over three years. At T3, a significant reduction in mean monthly headache days was evident, compared to T1 (3.4 ± 1.7 vs 7.2 ± 3.8; *P* < 0.001) with diminished mean number of monthly days with peak headache intensity of more than 4/10 and a significant change in days using acute headache medications per month between T1 and T3 (2.8 ± 1.3 vs 4.7 ± 3.2; P < 0.001). Significant changes were also noticed in all efficacy variables from T2 to T3. Therapy was safe and well tolerated with low rates of adverse events or drop-outs.

**Conclusion:**

The long -term treatment with onabotulinumtoxinA proved effective, safe and well tolerated over three years. Our findings support the strategy to consistently deliver sessions of use of onabotulinumtoxinΑ over long time in CM patients (Trial registration NTC03606356, registered retrospectively, 28 July 2018).

## Background

The clinical diagnosis of chronic migraine (CM) requires 15 or more headache days per month, of which at least 8 are of migrainous type or respond to migraine-specific medications, for more than 3 months [1]. In most cases, CM evolves from the episodic form of the disease, over a lengthy period of time. Given its debilitating nature, various pharmacological preventive treatments, including anticonvulsants, antidepressants and beta blockers have been tested, in an effort to reduce frequency and severity of attacks and intake of acute medication. However, clinical experience shows that a significant rate of patients remains refractory or intolerant to treatment after administering three or even more lines of prophylactic treatment [2].

ΟnabotulinumtoxinA (Botox®) is the only currently approved treatment for CM prophylaxis, following the publication of results from the Phase-III randomized placebo-controlled identical clinical trials PREEMPT (Phase III REsearch Evaluating Migraine Prophylaxis Therapy) I and II [3, 4]. Several other studies have subsequently been published on its use in CM. At present, there is a consensus that a response rate of about 65% may be expected after three courses of onabotulinumtoxinA therapy in CM patients [5, 6], but whether onabotulinumtoxinA may be continued after three sessions and for how long, has not been yet clarified.

Literature contains few studies on the real long-term experience with onabotulinumtoxinA in CM patients. The majority of those long-term studies have evaluated the results of up to two years of treatment [7, 8]. The recently published well-designed COMPEL study tested the long-term efficacy over two years of treatment [9]. In addition, there are two reports describing the outcome of long-term treatment during up to three and five years of administration, respectively [10, 11]. Overall, available data indicate that onabotulinumtoxinA may sustain its efficacy in CM prophylaxis for many years without significant adverse events [12].

We have previously reported that 3 courses of onabotulinumtoxinA prophylactic therapy were able to effectively reduce both the mean headache days/month as well as the days with peak headache intensity > 4/10, compared to baseline, in a cohort of 81 CM patients. A reduced intake of acute headache medications per month was also apparent. The treatment with onabotulinumtoxinA was safe and well tolerated [13]. We herein report the long-term therapeutic benefits of onabotulinumtoxinA against CM having followed the responders (> 50% reduction in mean headache days/month) of our core study sample who received treatment over three years. Our primary objective was to assess whether there is a sustained therapeutic effect of onabotulinumtoxinA in patients with CM after three years of treatment (months 37 to 39 after treatment initiation).

## Methods

### Study design, ethics, consent and permissions

The original study population belongs to an open-label, single-arm, prospective, observational clinical study that took place at five headache centres located in four different nodal geographic parts of Greece, including the major urban areas of Athens, Thessaloniki, Patras and the island of Corfu (Vikelis et al., 2016). Certified Botox injectors commenced treatment to each patient and continued administering treatment throughout the study’s period. The principal investigator’s Institutional Review Board (Mediterraneo Hospital, protocol no. 2719) granted approval for the current extension study, which was conducted in accordance with the principles of the Helsinki Declaration. Written informed consent for participation and publication was obtained from each patient before entering the current extension study.

### Intervention

Treatment with onabotulinumtoxinA (Botox® 100UI/fl, Allergan-Hellas) was administered according to the PREEMPT paradigm [3], as previously described in the core study [13]. Both in the core and current extension studies, administration of additional 40UI was allowed, in line with the PREEMPT “follow the pain” paradigm, at the injector’s discretion and according to individual patients’ needs, while intervals between the treatment sessions were allowed to be adjusted according to each patient’s needs, thereby individually potentially exceeding the 3-months period indicated by the PREEMPT paradigm.

### Patient selection

To be eligible for enrolment in the current setting, participants of our core study had to be classified as responders after 3 sessions of treatment, having experienced a ≥ 50% reduction in their average monthly headache days. [13]. All participants in our initial study had to be over 18 years of age at enrolment and diagnosed with CM with or without medication overuse. Patients were allowed to use acute symptomatic treatment as needed and in that case, they were asked to record it in their headache diaries.

### Efficacy evaluation

Primary and secondary objectives of this analysis remained the same as those of the core study and only the time points of evaluation were moved forward. Briefly, our primary goal was to assess the efficacy of onabotulinumtoxinA, as evaluated by the change in the mean number of monthly headache days, from the period after the third administration (months 10 to 12; T1) to the period after three years of treatment (months 37–39; T3). To fully monitor the time course of response, we also compared data obtained from the period after two years (months 25–27; T2) to T3.

Our secondary measures included the change in migraine severity as evaluated by the change in the number of days with peak headache intensity of more than 4 out of 10 in a 0–10 numerical scale (moderate / severe pain), as well as the change in days with any acute headache medication use between T1 and T3. The rest of our methods remained the same as previously described [13], in order to fulfil the current therapeutic and research plan requiring to administer onabotulinumtoxinA to each participant over three years before assessing long term efficacy. The reason for early discontinuations before T3 was also recorded.

### Safety evaluation

At each visit, adverse effects were recorded and then evaluated for potential relationship to onabotulinumtoxinA therapy, as previously described [13].

### Statistical analysis

Descriptive statistics were generated for all variables using the SPSS for Windows (release 17.0; SPSS Inc., Chicago, IL). The Wilcoxon rank test for paired data was used to assess potential changes in mean values of efficacy variables, between predefined time points, including T1 vs T3 and T2 vs T3. All tests were two-sided and significance was set at *P* < 0.05.

## Results

A significant proportion (86.1%, *n* = 56;) of patients who were classified as responders in the core study (*n* = 65), remained at onabotulinumtoxinA therapy for over three years, thus comprising the population of the analysis that we herein report. Table 1 summarizes the epidemiological characteristics and the clinical phenotype of our sample, whereas Table 2 reports the reasons for treatment discontinuation before T3 in 9 patients. Briefly, the majority (5 out of 9) of the drop-outs were due to significant improvement of the patients, who perceived that no additional sessions were needed, while some patients dropped out of the study due to financial limitations or they were lost to follow- up. As per protocol, not all of the patients had a treatment session every 3 months. A total of 16 patients continued treatment every 4 or 6 months (two or three sessions per year) after the first year of therapy rather than the typical 3-months interval between administrations. The decision for less frequent sessions was taken after discussion and agreement between the patient and the treating physician and it was based on a good response to the treatment in all cases.

**Table 1:** Patients’ baseline data and clinical characteristics

Variable	Study sample *n = 56* N %
Gender	
females	50 89.3
males	6 10.7
Age ± SD (range	43.3 ± 9.5 (21–60)
Previous lines of prophylactic medications	
Median value	3 (1–7)
Psychological comorbidities	
Anxiety	12 21.4
Depression	12 21.4
Anxiety and depression	10 17.9
Bipolar disorder	3 5.4
None	19 33.9

**Table 2:** Reasons for discontinuation of onabotulinumtoxinA treatment (*n* = 9)

Reasons for discontinuation of Onabotulinumtoxin-A treatment	N (%)
Lost to follow-up	3 (33.3%)
Financial limitations	1 (11.1%)
Patients significantly improved/ No additional sessions were needed	5 (55.5%)

T1, T2: Follow up after 9 and 24 months, respectively

The analysis of the primary response measure in the efficacy population (*n* = 56) showed that there was a further significant decrease in mean monthly headache days between T1 and over three years of therapy at T3 (7.2 ± 3.8– range: 2–15 vs 3.4 ± 1.7 – range: 1–11; *P* < 0.001). Likewise, the mean number of monthly days with peak headache intensity of more than 4 in a 1–10 numerical scale (moderate / severe pain) was also changed between T1 and T3 with a strong trend to significance towards further reduction (3.4 ± 2.8– range: 0–14 vs 2.5 ± 1.1 – range: 1–5; *P* = 0.052). As a result of the monthly headache frequency decrease along with the reduction of headache severity, we also recorded a significant change in the days of acute headache medication use per month between T1 and T3 (4.7 ± 3.2– range: 0–14 vs 2.8 ± 1.3 – range: 1–7; *P* < 0.001). Significant changes towards further improvement occurred in all efficacy variables from T2 (year 2) to T3 (year 3), thereby supporting the sustained efficacy of onabotulinumtoxinA consistently administrated in a long-term basis. Table 3 summarizes the longitudinal changes in all efficacy variables from baseline (T0 - trimester before initiation of therapy) over three years of onabotulinumtoxinA (T3) therapy. It should be noticed no patient in our group became resistant to onabotulinumtoxinA treatment.

**Table 3:** Longitudinal changes in all efficacy variables from baseline (T0 - trimester before initiation of therapy) over three years of onabotulinumtoxinA (Botox ®) (T3) in 56 patients

Efficacy Variables	T0 Mean ± SD Range Median	T1 Mean ± SD Range Median	T2 Mean ± SD Range Median	T3 Mean ± SD Range Median	*P* value ^*^T1 vs T3 ^**^T2 vs T3
Headache days/month	21.5 ± 5.1 14–30 20	7.2 ± 3.8 2–15 6	5.4 ± 2.6 1–11 5	3.4 ± 1.7 1–11 4	^*^P < 0.001 ^**^P < 0.001
Number of days with peak headache intensity of more than 4/10 per month	11.7 ± 5.7 4–30 10	3.4 ± 2.8 0–14 3	3.2 ± 1.6 1–7 3	2.5 ± 1.1 1–5 2	^*^P = 0.052 ^**^P < 0.001
Days with any acute headache medication / month	16.5 ± 7.3 7–30 14	4.7 ± 3.2 0–14 4	3.4 ± 1.7 1–8 3	2.8 ± 1.3 1–7 2	^*^P < 0.001 ^**^P < 0.001

T0 refers to the trimester before initiation of therapy; τ1 refers to the trimester after the third onabotulinumtoxinA administration, T2 to the trimester after completing two years of treatment and T3 to the trimester after completing three years of treatment

*P values stand for significant changes between T1 vs T3

**P values stand for significant changes between T2 vs T3

A total of 50 out of the 56 patients that completed 3 years of treatment in this study continued treatment with onabotulinumtoxinA (additional sessions performed beyond month 36) and we plan to publish results of even lengthier treatment in a future publication.

The safety analysis showed that the onabotulinumtoxinA treatment was safe and well tolerated, without severe side effects justifying treatment discontinuation. However, there were a few cases experiencing transient and mild adverse events, including wheals in the injection site and shoulder and/or neck pain, at comparable rates with those of the core study [13].

## Discussion

At present, onabotulinumtoxinA has established its efficacy as a prophylactic intervention in CM for at least one year of therapy [14]. However, given that CM patients often relapse after discontinuation of treatment, especially in presence of medication overuse [15], it is important, from a clinical point of view, to further ascertain if there is a sustained benefit for CM patients in the long term after at least three years of treatment with onabotulinumtoxinA. The latter becomes even more important as new treatment options for chronic migraine are anticipated in the near future, including monoclonal antibodies against CGRP or its receptor or small molecules targeting CGRP [16] and our study deals with this issue, providing long term efficacy and safety data, derived from an observational, real-world, multicentre setting in Greece. In our knowledge, there is no similar study so far to report on the long-term outcome of onabotulinumtoxinA intervention in a population of Greek patients with CM.

As already mentioned, we have previously reported that three sessions of onabotulinumtoxinA as a prophylactic therapy were able to effectively reduce both the frequency and the severity of CM, compared to the baseline. A consequent reduction of acute headache medication intake was also observed [13]. After successfully completing 3 cycles of treatment, patients were offered to continue treatment or not. All our patients had a history of chronic migraine and failed one or more oral prior preventives [13] and the decision of most of them to continue a successful treatment was no surprise. With our present results, we document a sustained long term therapeutic benefit with the prophylactic use of onabotulinumtoxinA against CM over three years of treatment.

Noteworthy, we recorded a further reduction in the mean monthly headache days along with reduced severity of headache and decreased acute medication intake over the first two years (months 25–28) and even beyond three years (months 37–39) of treatment, compared to the period after 3 treatment sessions (T1; months 6 to 9). This finding strongly supports a suggestion to regularly repeat sessions with onabotulinumtoxinA beyond the first year of treatment in order to obtain a sustained therapeutic benefit and to avoid relapse in case of treatment discontinuation [17].

Interestingly, despite the fact that relapse rates are high after CM treatment, especially in the presence of co-morbid medication overuse headache, in many cases recommendations insist on weaning or even discontinuing treatment after a few months or one year. This has been commonly recommended for orally given preventive medications [18], presumably due to the possibility of adverse events after long term use. Although there is no consensus on management of responders to onabotulinumtoxinA treatment [19] the strategy of discontinuing treatment after initial response is often applied with onabotulinumtoxinA treatment. As clinical data accumulate and additional knowledge on CM pathophysiology is gained [20], we consider possible that future recommendations will suggest long term treatment for CM patients regardless of the intervention used, provided, of course, that treatment is efficacious and adverse events are insignificant.

Our findings on the long term beneficial effects of onabotulinumtoxinA, overall, are in agreement with previously published studies that have also continued treatment for two to five years [9–11], although methodological differences between those studies do not allow for confident comparisons. For example, in contrast to the COMPEL study [9], no participant in our study used any concomitant medication with a prophylactic effect on CM, with the exception of antidepressants prescribed to treat psychiatric comorbidities. Furthermore, contrary to other previously published studies dealing with the same topic, we have applied a prospective, real-world data study design, rather than a retrospective analysis [10, 11, 21]. It is noticeable that no patient in our group became resistant to treatment. The relatively small overall number of patients may account for this.

Current knowledge derived from clinical practise shows a low adherence to orally given preventive treatments due to intolerance [22]. This might also be the case in the long term onabotulinumtoxinA -treated patients, however, the results of our study dispute it. The majority of patients (86.1%) responding well after three sessions of onabotulinumtoxinA treatment continued treatment over three years. There is certainly a selection bias in our population, as all participants has been responders to an initial round of three treatment sessions, but in a real life clinical setting this finding both sheds further light on the excellent tolerance and safety profile of onabotulinumtoxinA in the long term-treatment of CM, and underscores the relative low rates of treatment discontinuation. As additional data on long term use of onabotulinumtoxinA in CM accumulate from larger studies, as the REPOSE study that has enrolled more than 600 patients from seven European countries with a treatment plan of two years [23], we anticipate with great interest if our results shall be reproduced.

One might claim that a different study design could provide even more robust results than our open-label, single-arm study without placebo control. However, we have chosen this design as optimal, in a similar way with the COMPEL study [9], considering the extended duration over 36 months and the established efficacy and safety of onabotulinumtoxinA therapy in CM for up to one year. The lack of placebo exposure over long time might have also contributed to the diminished discontinuation rates.

As a closing remark, we can state that the current study adds on the body of existing literature as it reports the outcome of an observational, real-world data study in Greece that aimed to prospectively assess the long-term effects of onabotulinumtoxinA against CM, extending the available data on its efficacy and safety profile when given for at least three years.

## Conclusions

The long-term treatment with onabotulinumtoxinA proved effective, safe and well tolerated over three years. Our findings demonstrate further improvement in all efficacy variables from months 6–9 to months 25–28 and even a more marked improvement at months 37–39, supporting the suggestion of routine repeated administration of onabotulinumtoxinA over long time in CM patients. Accumulative clinical experience, including the present one, establishes the very significant role that onabotulinumtoxinA possesses in CM treatment with sustained therapeutic benefit over long time. On clinical grounds, we strongly believe that onabotulinumtoxinA will continue to have a pivotal role in CM therapy.

## Abbreviations

CGRP: Calcitonine Gene Related Peptide
CM: Chronic Migraine
